# Fluorine-19 nuclear magnetic resonance of chimeric antigen receptor T cell biodistribution in murine cancer model

**DOI:** 10.1038/s41598-017-17669-4

**Published:** 2017-12-18

**Authors:** Fanny Chapelin, Shang Gao, Hideho Okada, Thomas G. Weber, Karen Messer, Eric T. Ahrens

**Affiliations:** 1 Department of Bioengineering, University of California San Diego, San Diego, CA USA; 2 Department of Radiology, University of California San Diego, San Diego, CA USA; 3 Department of Neurological Surgery, University of California San Francisco, San Francisco, CA USA; 4 Cancer Immunotherapy Program, University of California San Francisco, San Francisco, CA USA; 5 Cancer Prevention and Control Program, Moores Cancer Center, University of California San Diego, La Jolla, CA USA

## Abstract

Discovery of effective cell therapies against cancer can be accelerated by the adaptation of tools to rapidly quantitate cell biodistribution and survival after delivery. Here, we describe the use of nuclear magnetic resonance (NMR) ‘cytometry’ to quantify the biodistribution of immunotherapeutic T cells in intact tissue samples. In this study, chimeric antigen receptor (CAR) T cells expressing EGFRvIII targeting transgene were labeled with a perfluorocarbon (PFC) emulsion *ex vivo* and infused into immunocompromised mice bearing subcutaneous human U87 glioblastomas expressing EGFRvIII and luciferase. Intact organs were harvested at day 2, 7 and 14 for whole-sample fluorine-19 (^19^F) NMR to quantitatively measure the presence of PFC-labeled CAR T cells, followed by histological validation. NMR measurements showed greater CAR T cell homing and persistence in the tumors and spleen compared to untransduced T cells. Tumor growth was monitored with bioluminescence imaging, showing that CAR T cell treatment resulted in significant tumor regression compared to untransduced T cells. Overall, ^19^F NMR cytometry is a rapid and quantitative method to evaluate cell biodistribution, tumor homing, and fate in preclinical studies.

## Introduction

Immunotherapy, using engineered T cells harboring receptors targeting specific tumor antigens, has opened the path to new treatments for incurable cancers^1^. Cancer cells secrete cytokines that render the host’s innate and adaptive immune system ‘tolerant’ to the tumor, which weakens the intrinsic immunity^2^. In an emerging approach, autologous T cells are genetically modified *ex vivo* to constitutively express a chimeric antigen receptor (CAR) that can help bind T cells to a specific tumor target and overcome tolerance. By delivering high numbers of CAR T cells and stimulating their clonal expansion *in situ*, specific and durable cytotoxicity toward cancer cells has been observed^3^. This strategy has been translated to many types of cancers including leukemia, lymphomas and sarcomas, with custom made receptors for each application^4–6^.

Adoptive cell cancer therapy is currently being used in at least 270 active clinical trials worldwide^7^. However, variability in clinical outcomes and the incidence of harmful side effects has challenged researchers to implement methods to validate cell biodistribution and pharmacokinetics in the body^3^. In fact, the United States Food and Drug Administration (FDA) published guidelines^8^ specifying that investigational cell therapy should incorporate some means of cell tracking to determine *in vivo* cell survival, anatomic engraftment and biologic activity throughout the product development cycle, preferably starting at the preclinical stage. Indeed, the current gold standard to assess cell biodistribution preclinically involves time-consuming necropsy and histopathological staining of numerous tissue slices, which, in addition to being tissue-disruptive, only provides quantitative cell information on small tissue ‘bites’ which is prone to sampling error. Developing a rapid and quantitative preclinical technique for screening new therapeutic cell subtype candidates by assessing cell biodistribution and survival would be highly useful.

Here, we describe the use of nuclear magnetic resonance (NMR) ‘cytometry’^9^ to assay immunotherapeutic cell biodistribution. This technology employs a perfluorocarbon (PFC) nanoemulsion tracer that labels cells via simple co-incubation in culture prior to *in vivo* delivery. Liquid-state ^19^F NMR spectroscopy of intact, excised organ and tissue panels is used to measure the effective number of transferred cells within each sample^10–12^. Consequently, the cell biodistribution and survival can be rapidly measured, and specific T cells homing to the tumor and lymphoid organs can be measured, which is presumably predictive of a positive clinical response. We employ a murine model of subcutaneous human glioblastoma treated with CAR T cells expressing Epidermal Growth Factor Receptor variant III (EGFRvIII) transgene^13,14^. In solid tumors, EGFRvIII is a common tumor-specific variant associated with poor long-term survival^15^. EGFRvIII is present in ~20% of glioblastoma multiforme (GBM) patients; GBM is the most common and aggressive brain cancer^16,17^. Prior to CAR T cell infusion, the cells are intracellularly tagged with PFC emulsion *in vitro*, and the ^19^F labeling efficiency, cellular function, and phenotype is verified post-labeling. Following infusion, CAR T cell efficacy is monitored by bioluminescence imaging (BLI) *in vivo*, and CAR T cell biodistribution and pharmacokinetics are quantitated using *ex vivo* ^19^F NMR. Overall, NMR cytometry may accelerate the timeline to evaluate and screen new engineered cell therapeutic candidates.

## Results

### *In vitro* characterization of CAR-expressing T cells

**Figure 1:**
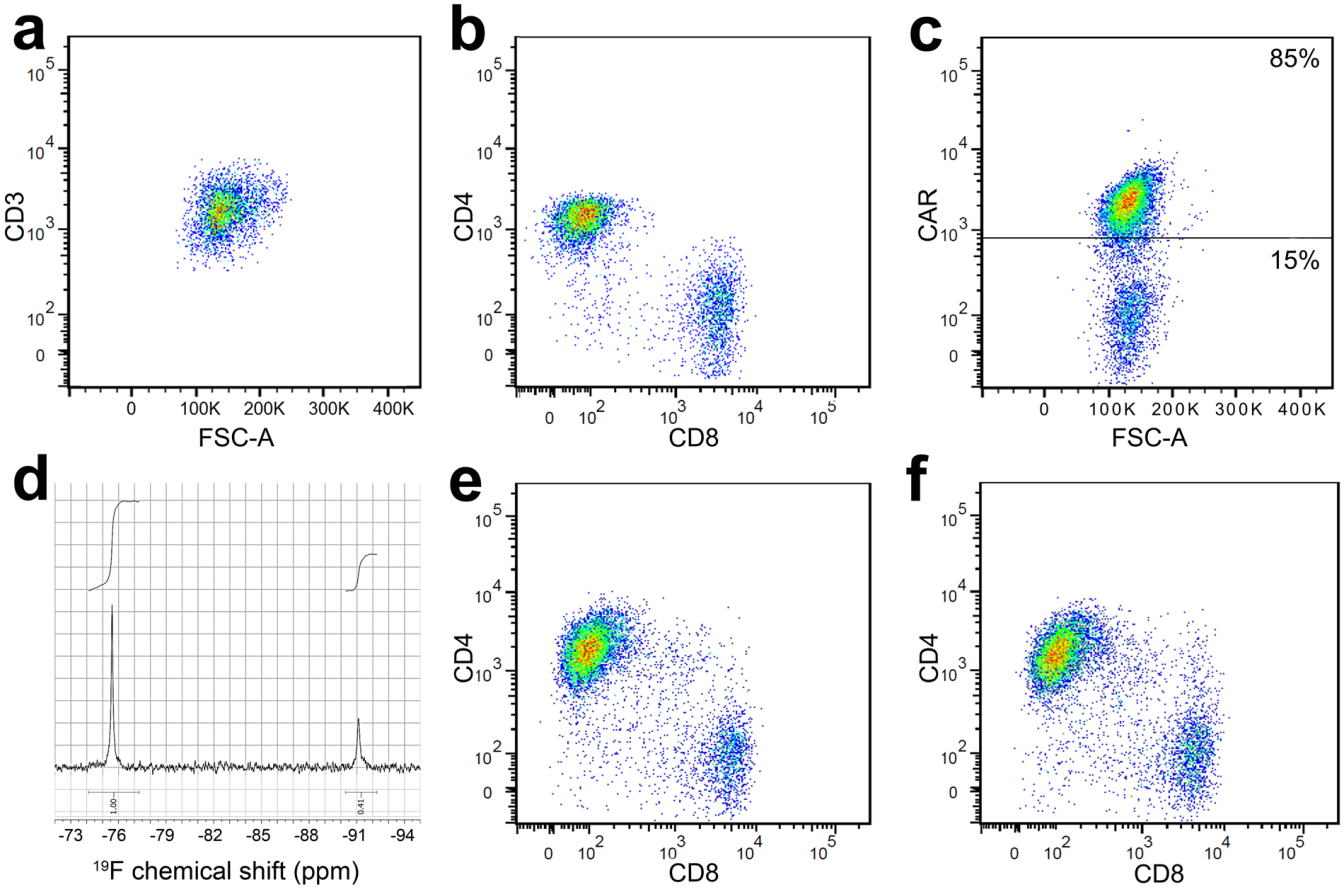
CAR T cell transduction and characterization. (**a)** Scatter plot showing the pure population of human T cells (CD3) after magnetic assisted cell sorting of blood samples. (**b**) Isolated T cell flow analysis for expression of CD4/CD8 shows that 2/3 of T cells are CD4+ and 1/3 are CD8+. (**c**) CAR T cell population 2 weeks after transduction shows 85% CAR-expressing T cells. (**d**) ^19^F NMR spectrum showing PFC uptake of CAR T cells (peak at −91 ppm, 2 × 10^11^ atoms/cell) normalized to the TFA reference (peak at −76 ppm). (**e**) Flow cytometry histogram showing similar repartition of CD4+ and CD8+ CAR T cells after transduction compared to untransduced T cells (**b**). (**f**) CAR T cells labeled with PFC *ex vivo* exhibit comparable phenotype to unlabeled cells.

Initially, we assessed the phenotype and PFC labeling levels in T cells. The lymphocyte isolation from PBMC yields a pure population of CD3+ T cells with an approximate 2/3 CD4+ and 1/3 CD8+ phenotype distribution (Fig. 1A and B). In T cells transduced with lentivirus harboring EGFRvIII antibody, transgene expression levels persist, with >70% of the human T cells expressing the CAR receptor after two weeks *in vitro* (Fig. 1C). For *in vivo* animal studies (below), infused T cells were 85 ± 10% CAR-positive.

Labeling experiments with PFC nanoemulsions at 10 mg/ml over a period of 12 hours co-incubation display minimal viability impairment as assessed by Trypan blue exclusion test (Average 95 ± 1%, N = 3 replicates) and flow cytometry viability measurements (Supplementary Table 1, p > 0.05). These conditions yield an average labeling efficiency of 2 ± 0.5 × 10^11^ atoms of fluorine per cell (N = 3 replicates, Fig. 1D), as determined by ^19^F NMR. Moreover, PFC labeling does not appear to alter T cell phenotype as defined by CD4+ and CD8+ expression or cell proliferation (Fig. 1E and F and Supplementary Table 1, p > 0.05).

**Figure 2:**
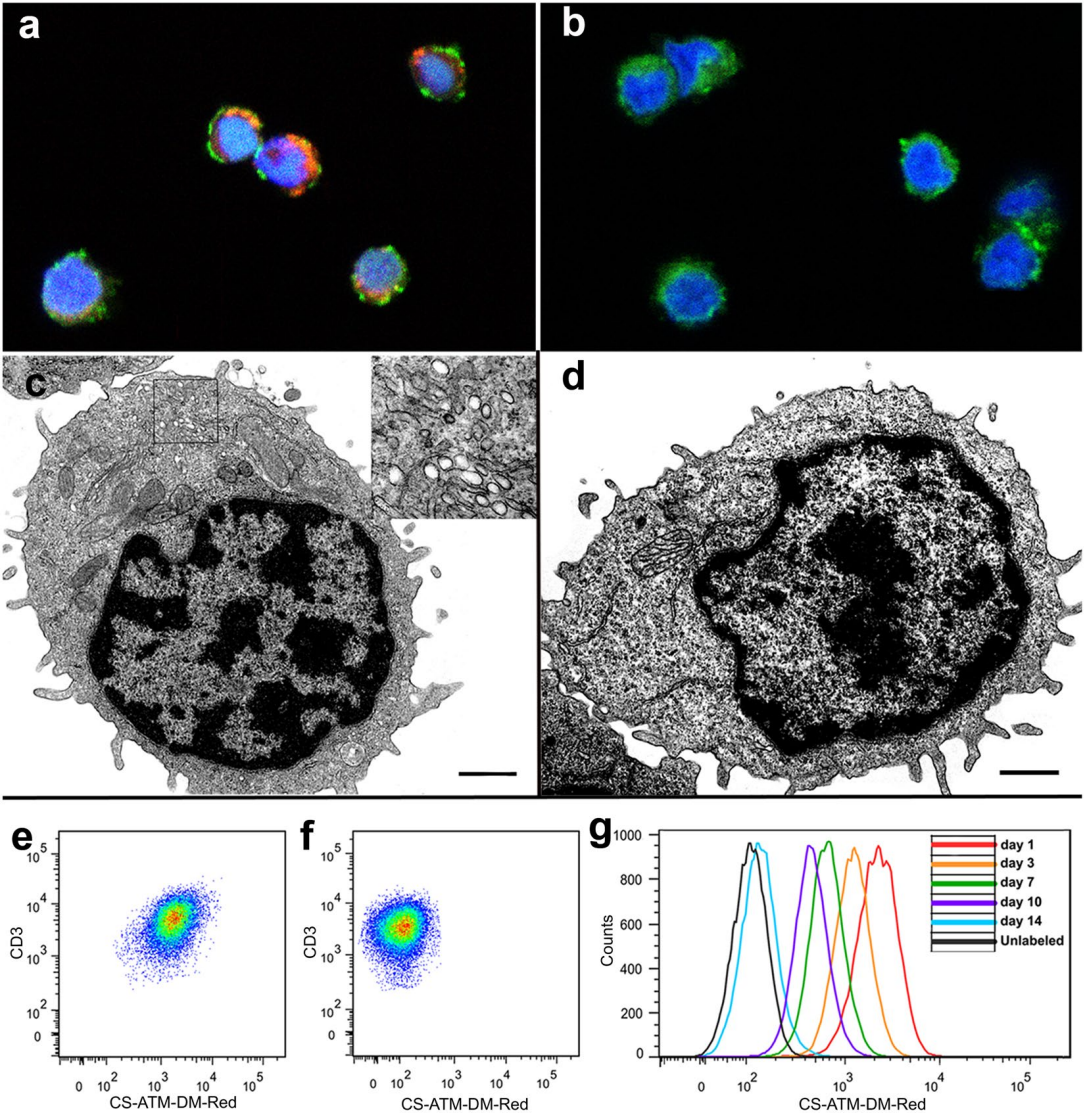
Confirmation of intracellular localization of PFC. (**a**) Confocal image (×63) showing perinuclear localization of dual-mode PFC nanoemulsion (in red) in CAR T cells. Nucleus is stained in blue and cell membrane in green via CD3-FITC (**b**) Control unlabeled CAR T cells do not exhibit red fluorescence. (**c**) Electron microscopy (EM) micrograph of ultra-thin PFC-labeled CAR T cells reveals the presence of bright vesicles (inset = ×11,000) corresponding to internalized PFC droplets. (**d**) In control unlabeled cells, no PFC vesicles are found (×4800, scale bars = 1 μm). (**e**) Flow cytometry scatter plot showing the resulting shift in red fluorescence of CAR T cells after labeling. All cells are labeled after overnight incubation as evidenced by fluorescence gap between labeled (**e**) and unlabeled (**f**) CAR T cells. (**g**) Longitudinal red fluorescence measurement after labeling shows persistent signal and consistent decrease of fluorescence intensity due to cell division.

Intracellular and perinuclear localization of PFC label in CAR T cells was confirmed by confocal microscopy (Fig. 2A,B) using a dual-mode MRI-fluorescent PFC nanoemulsion. This result was corroborated by electron microscopy (Fig. 2C,D). Bright spheroids corresponding to electron-sparse scattering perfluorocarbon droplets were found in most labeled cells (Fig. 2C, see inset, ×11,000) but not in control unlabeled cells (Fig. 2D). Flow cytometry (Fig. 2E,F) measurement of CAR T cells labeled with the dual mode agent revealed a shift in red fluorescence compared to control unlabeled cells indicating that essentially 100% of CAR T cells are labeled with PFC one day after labeling (Fig. 2E). The fluorescence shift remains discernable for several days after labeling but returns to unlabeled baseline by day 14 due to cell division and agent dilution; the degree rate of label dilution is consistent with the CAR T cell division rate *in vitro*.

### CAR T cells exhibit cytotoxicity against U87-EGFRvIII glioma cells

**Figure 3:**
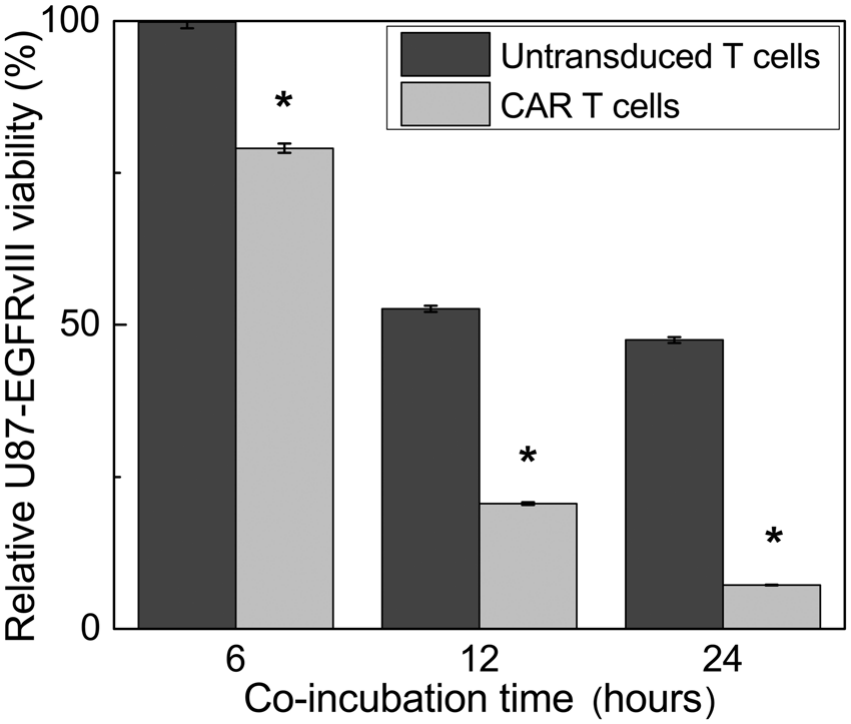
Human CAR T cell cytotoxicity assay *in vitro*. Co-incubation of CAR T cells or untransduced T cells with human U87-EGFRvIII-Luc glioma cells resulted in significant cell death at 6, 12 and 24 h. CAR T cells exhibit significant tumor killing ability (93%) compared to untransduced T cells (53%, p < 0.0001 indicated by *). The relative glioma cell cytotoxicity was obtained from the mean photon count, and the ratio of treated versus untreated means are displayed as percentages ± standard error.

CAR T cells exhibit significant, antigen specific, cytotoxic activity against U87-EGFRvIII-Luc cells *in vitro* (Fig. 3). Ultraviolet-visible (UV-Vis) spectroscopy measurements show that CAR T cells induce 93% tumor cell death compared to 53% for untransduced T cells at 24 h (Fig. 3). These results are statistically significant at all time points (p < 0.0001). Untransduced T cells generated notable non-specific tumor cell death, but apoptosis induction plateaued rapidly to 50%. Pro-inflammatory cytokines present in the media, including IL-2 and CD3/CD28 beads, may have contributed to untransduced T cell activation, leading to granzyme and perforin release and detectable cancer cell death^18^. Nonetheless, these *in vitro* results confirmed the targeted cytotoxic activity of CAR T cells against EGFRvIII + glioma cells.

### CAR T cells inhibit tumor growth

**Figure 4:**
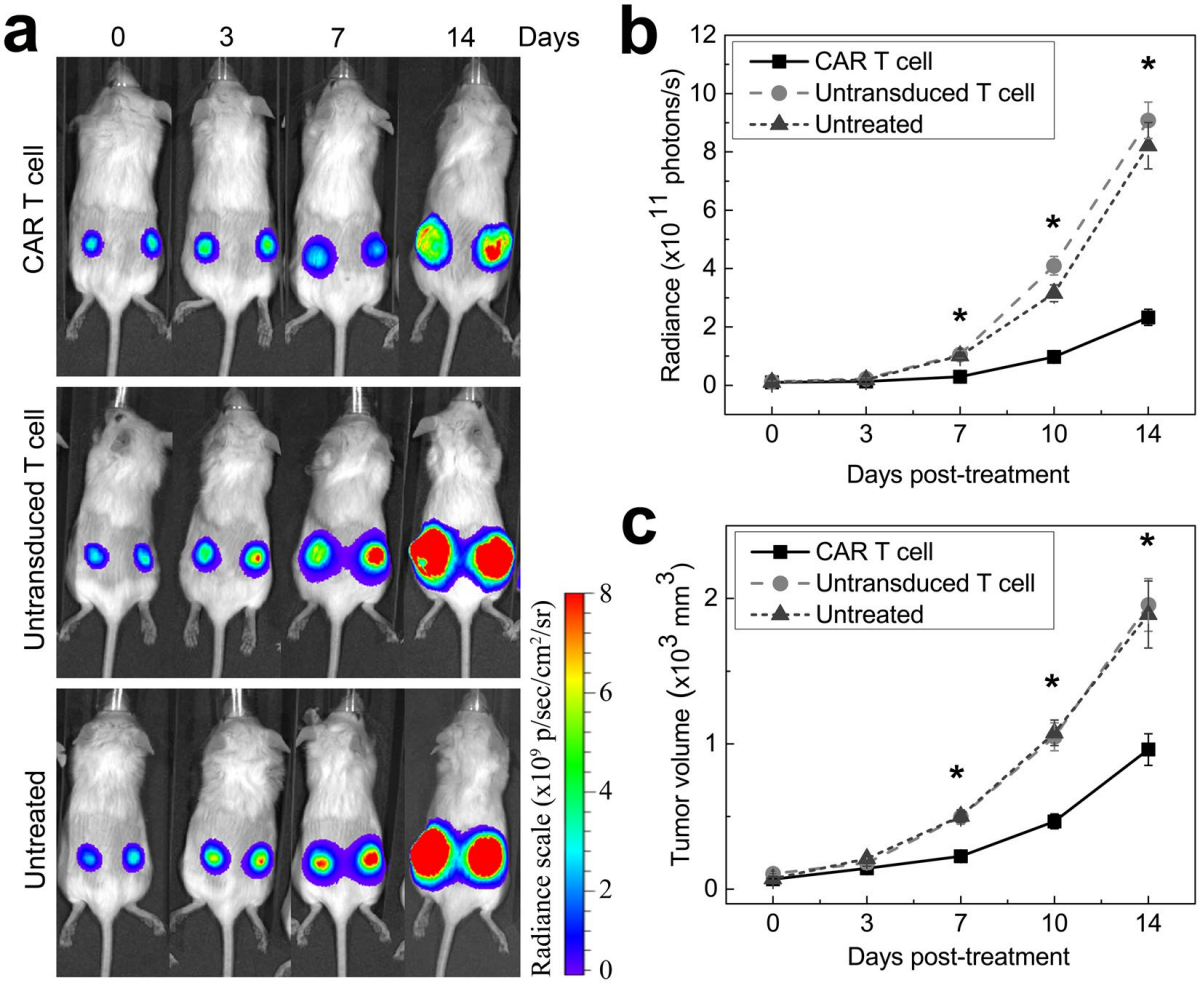
Impact of cell therapy on tumor growth *in vivo*. (**a**) Representative bioluminescence images at day 0, 3, 7, 10 and 14 after cell therapy for all three groups show tumor growth reduction for the CAR T cell treated mice compared to untransduced T cell treated or untreated mice. (**b**) Bioluminescence signal is significantly lower in the CAR T cell treated group starting at day 7 compared to controls. CAR T cell treated animals display a radiance four times lower than untransduced T cell treated animals or untreated animals at day 14 (*indicates p < 0.0003 at day 7, 10 and 14). (**c**) Corresponding tumor volume measurements show 50% reduction in tumor growth for the CAR T cell treated group at day 7, 10 and 14 (*indicates p < 0.0003) and no significant difference between untransduced T cell treated and untreated groups (p = 0.38).

The anti-tumor efficacy of PFC-labeled CAR T cells was verified using immunocompromised SCID mice bearing bilateral subcutaneous U87-EGFRvIII tumors. Engraftment of tumors was confirmed by BLI and caliper measurements just prior to T cell infusion (day 0). Longitudinal BLI and tumor measurements show considerably reduced tumor growth as early as 7 days after CAR treatment (Fig. 4A) compared to controls. The radiance of CAR-treated tumors average 2.9 ± 0.4 × 10^10^ photons/s, which was three times lower than the luminescence for both the untransduced T cell treated (1.06 ± 0.1 × 10^11^ photons/s) and untreated (1.01 ± 0.1 × 10^11^ photons/s) groups (Fig. 4B, F-statistic F(2,29) = 18.06, p < 0.0003). Similarly, the mean tumor volume at day 7 in the CAR-treated group is 226 ± 28 mm^3^, compared to 497 ± 50 mm^3^ and 502 ± 41 mm^3^ for untransduced T cells and untreated groups, respectively (Fig. 4C, p < 0.003). The disparity among groups grew until the last measurement on day 14, where the mean luminescence in the CAR-treated group is 2.33 ± 0.3 × 10^11^ photons/s, and the untransduced T cell and untreated groups is 9.08 ± 0.6 × 10^11^ and 8.21 ± 0.8 × 10^11^ photons/s, respectively. The T-test p-value between CAR treated and untransduced T cell treated or untreated is ≤0.0001; the corresponding ANOVA test of the three groups F(2,29) = 18.06–37.29, p < 0.0003 is extremely significant at 7, 10 and 14 days post-treatment, using a Bonferroni correction.

**Figure 5:**
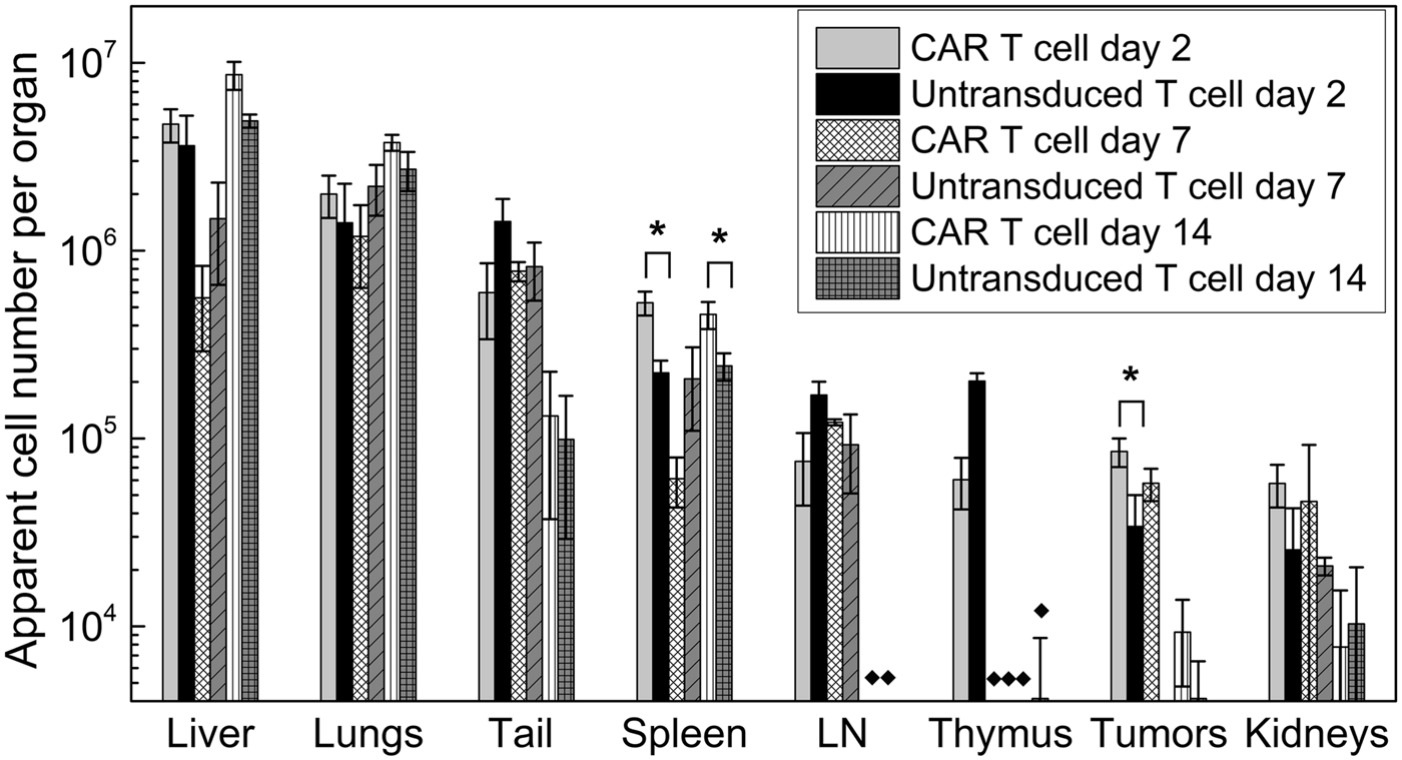
Biodistribution of tissue samples by 19F NMR at 2, 7 and 14 days post-treatment. ^19^F NMR measurements of organ biodistribution of the PFC-labeled cells show greater CAR T cell homing to the spleen and tumors compared to untransduced T cells at day 2. The liver signal likely represents the dead T cell fraction. These measurements do not account for cell division, thus referred to as ‘apparent’ cell number. LN indicates lymph nodes, (*) indicate significance, and (♦) indicate pooled samples.

The cancer cells in a solid tumor, in particular in the necrotic center, are much less accessible to the T cell pool compared to the *in vitro* case where the cancer cells are directly in contact with T cells. In fact, cell quantitation shows that the number of cells reaching the tumor *in vivo* is fairly low, and this is one of the plausible explanations as to why the CAR T cells fail to completely ‘reverse’ tumor growth (Fig. 5).

### ^19^F NMR cytometry of T cell biodistribution

NMR measurements on intact tissue samples enable quantification of the total ^19^F content and the apparent number of labeled T cells infiltrating the tissue (Fig. 5). The results at day 2 post-treatment show greater CAR T cell homing to the tumors and spleen compared to untransduced T cells (p = 0.01 and 0.04, respectively). Approximately 85,000 ± 14,000 CAR-T cells are found in the tumors at day 2. The number of apparent CAR T cells in the tumor is stable until day 7, whereas untransduced T cells are below the LOD (p = 0.0022), which is estimated to be on the order of ~7 × 10^3^ cells per sample for the 5 mm probe and ~4 × 10^4^ for the 10 mm probe (see Supplementary Fig. S1). On average, 5 ± 0.7 × 10^5^ CAR T cells (*i.e*., the equivalent amount of ^19^F signal) are found in the spleen at day 2 and 14, whereas only half this amount is observed in untransduced T cell treated animals. These NMR cytometry measurements do not account for possible T cell division *in vivo*; cell division can diminish the accuracy of the apparent total cell count per tissue, particularly at later time points.

The average ^19^F signal in the liver is approximately 15% of the injected dose at day 2 in both groups. At day 14, 33% of the fluorine signal is found in the liver. The liver ^19^F signal (Fig. 5) presumably represents the dead T cell fraction, as PFC droplets from dead cells are taken up predominantly by Kupffer cells of the liver^19^. No significant differences in ^19^F signal are found in the lung, lymph node, thymus and kidney between groups (Fig. 5) and therefore may be marginal contributors to the therapeutic effect. The tail ^19^F signal (Fig. 5) represents the fraction of cells that are ‘mis-injected’ during tail vein delivery and presumably resides in local subcutaneous tissue; using this measured value one can in principle calculate the actual T cell dose delivered intravenously in each subject by subtracting out this missed cell fraction. We speculate that the gradual decrease in signal from tissues/organs such as the lymph nodes and thymus is due to T cell death and is responsible for the increase in the liver signal at later time points. All measurements presented are above our LOD of ~7 × 10^3^ to ~4 × 10^4^ cells per sample (for 5 mm and 10 mm probes, respectively) for a 20 min acquisition time (see Supplementary Fig. S1). Organs with undetectable fluorine levels in all animals are not displayed (brain, heart, small intestine, spinal cord, femur, blood). In the recovered blood samples, the ^19^F NMR displayed signal below the LOD, thus residual blood in tissue samples would not be a significant source of false-positive signal.

### Histopathology

**Figure 6:**
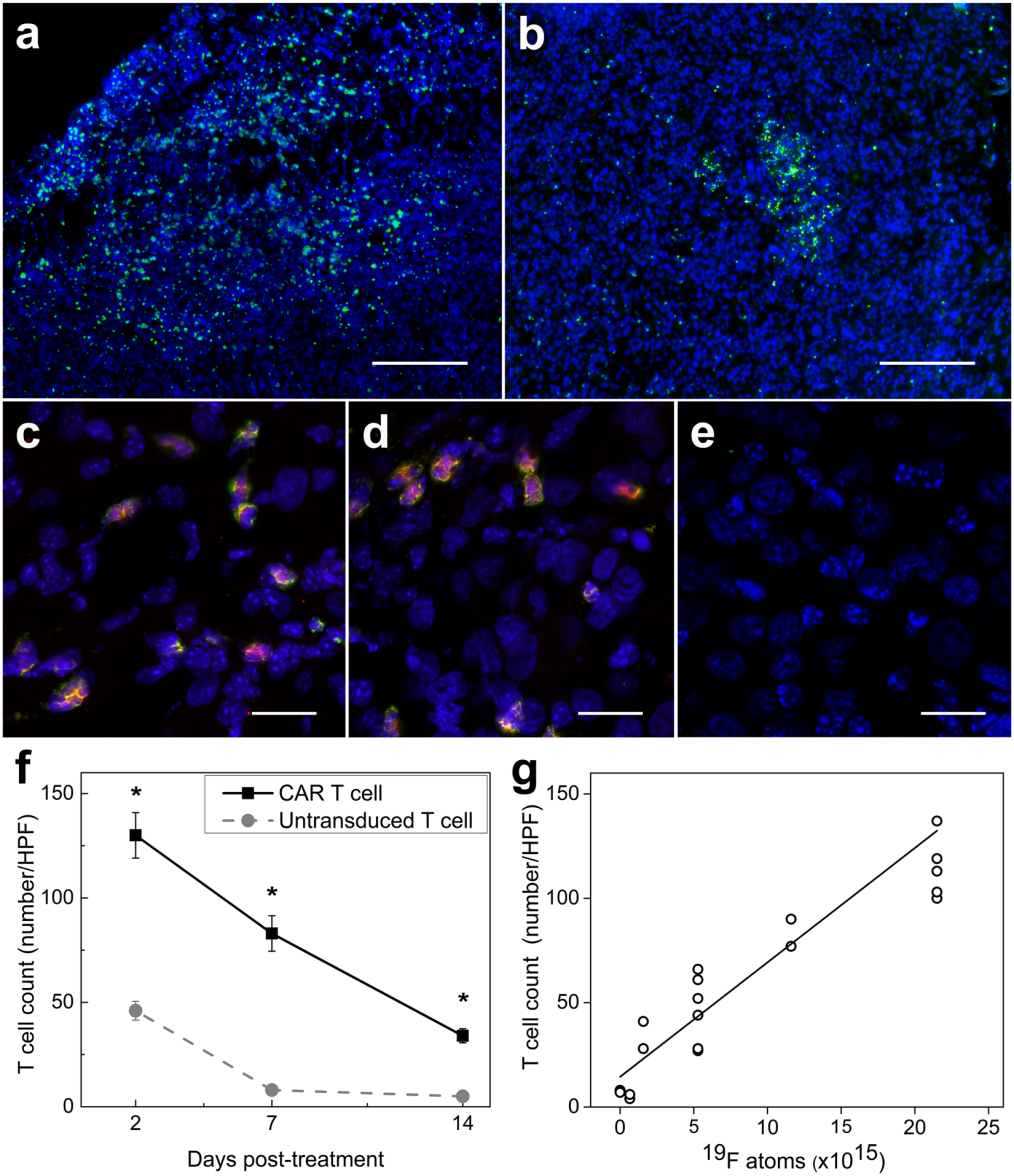
Histological correlation of tumor tissue of T cell treated SCID mice. (**a**) Low magnification (×5) fluorescent image of the tumor tissue displays prominent CD3 + CAR T cell infiltrates (green). (**b**) Conversely, sparse untransduced T cells localize to the tumor (scale bars = 100 μm). (**c**). Confocal images (×63) show that dual-mode PFC nanoemulsion (red) remains colocalized within CAR T cells (green, **c**) and untransduced T cells (**d**) at day 2 after infusion. (**e**) Untreated mice do not exhibit green or red fluorescence due to absence of treatment and endogenous T cells in SCID mice (scale bars = 25 μm). (**f**) Histological T cell count in high power fields of tumor sections shows greater CAR T cell numbers at all time points compared to sections from untransduced T cell-treated animals (*indicates p < 0.001). (**g**) Plot showing strong positive correlation (r = 0.89) between average ^19^F atoms in tumors as measured by NMR and T cell count (N = 120, HPF = high power fields).

**Figure 7:**
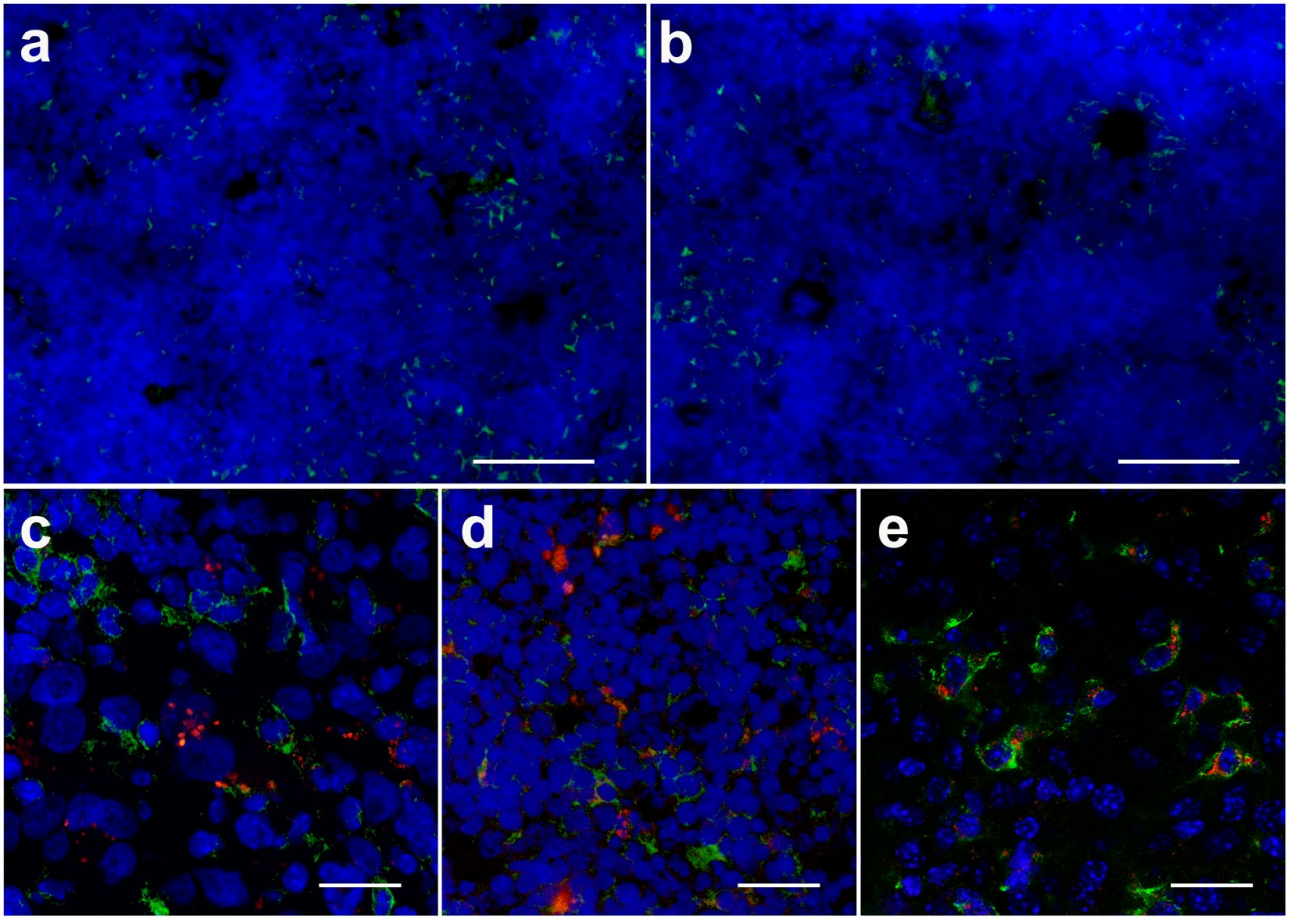
Histology in spleen and liver tissue of T cell treated SCID mice. (**a**) Low magnification (×5) fluorescent image of the spleen tissue displays prominent CD3 + CAR T cell infiltrates (green). (**b**) Untransduced T cells also localize to the spleen (scale bars = 50 μm). (**c**). Confocal images (×63) show that dual-mode PFC nanoemulsion (red) does not colocalize within phagocytic cells (F4/80 staining, green) in the tumor (**c**) and spleen (**d**) at day 2 after infusion. (**e**) Conversely, F4/80 staining in the liver tissue displays dual-mode PFC (red) colocalizing with Kupffer cells or liver macrophages (scale bars = 25 μm).

Histopathological staining confirms the presence of numerous CAR T cell infiltrates in the tumors as evident by the fluorescent green signal targeting human CD3 (Fig. 6A). In comparison, untransduced T cell infiltrates are sparser in these tissues (Fig. 6B). At day 2, both CAR-T cells and untransduced T cells show persisting red fluorescent signal corresponding to the dual-mode (CS-ATM DM Red) PFC nanoemulsion (Fig. 6C,D). No endogenous T cells are present in untreated SCID mice (Fig. 6E). Quantitative analysis of the number of CAR or untransduced T cells per high power field show significantly higher CAR T cell numbers in the tumor tissue than untransduced T cells at all time points (Fig. 6F). In addition, histology counts strongly correlate to the average NMR fluorine signal measured in tumors (Pearson’s r = 0.89, Fig. 6G). CAR T cells and untransduced T cells are also present in large numbers in the spleen (Fig. 7A,B). Red fluorescence does not colocalize to phagocytic cells in the tumor and spleen (Fig. 7C,D), which indicates minimal contamination of dead T cell signal in these tissues, contrary to phagocytic Kupffer cells in the liver (Fig. 7E), which contain red fluorescence signal from dead T cells.

## Discussion

We describe the use of NMR cytometry for quantifying the apparent biodistribution of human CAR T cell therapy in a murine xenograft model of glioma. We compared CAR T cell biodistribution and efficacy to untransduced T cells. Tumor growth and volume was assessed by bioluminescence imaging and caliper measurements. NMR measurements in whole organs demonstrated significantly higher CAR T cell homing to tumors and spleen compared to untransduced T cells, consistent with conventional histopathology staining. Our results corroborate the importance of the EGFRvIII receptor as a target for immunotherapy and the efficacy of CAR T cells against these tumors. Injection of untransduced lymphocytes did not result in enhanced tumor inhibition over untreated mice and appeared to have a lower and more transient accumulation at tumor sites compared to CAR T cells.

Our view is that NMR cytometry is an option for discovery and preclinical evaluation of emerging immunotherapeutic cell candidates by helping to streamline the evaluation of cell biodistribution and survival. One of the key advantages is that the analysis is in intact tissues, with the only treatment being an optional fixation step. PFC nanoemulsion internalized in cells does not alter the PFC chemical shift of the NMR spectrum which is defined by their molecular structure^20–22^. Moreover, the PFC label in tissue T cells is stable and can be stored or frozen indefinitely without signal loss^23–25^. Measurement of ^19^F content of a sample can be accomplished using conventional NMR spectroscopy in tens of minutes or less, and data acquisition can be automated with robotic sample changers and auto-shimming. Programing automated data analyses routines to extract the PFC ^19^F peak integrals and apparent cell counts is straightforward. Generally, NMR instrumentation is a routine analytic tool for molecule structure elucidation and is commonplace in virtually every major chemistry lab, with tens of thousands of such instruments sited worldwide. Use of *in vitro* NMR cytometry as described herein has the advantage of higher sensitivity to sparser cell numbers compared to MRI by an order magnitude^9^. We note that the use of preclinical ^19^F MRI in murine models of CAR T cell therapy remains a topic of future study.

If cell division occurs, the accuracy of the absolute T cell counts for NMR cytometry may diminish, particularly at later time points, thus we describe these as ‘apparent’ cell counts. The impact of cell division on PFC labeled T cell quantification during longitudinal studies in mouse is described in detail elsewhere^26^. Potentially a carboxyfluorescein succinimidyl ester (CFSE) assay can be used to determine mitosis rates *in vivo* ^27^. However, considering the heterogeneity of cell phenotypes used in CAR T cell therapies, as well as differential division rates in different tissues, estimation of whole-body CAR T cell division rates *in vivo* remains challenging.

T cells have weak phagocytic properties and a small cytoplasmic volume, thus are more challenging to label compared to larger phagocytic cells such as DCs. Nonetheless, authors have successfully labeled T cells *in vitro* using iron-based nanoparticles^28^ and fluorinated nanoemulsions^29,30^. The PFC nanoemulsion used in this study (CS-1000) has previously been used for numerous preclinical ^19^F magnetic resonance imaging (MRI) studies of immune and stem cells in various disease models^23,30–38^. By means of fluorescence, electron microscopy and/or flow cytometry, dual-mode PFC nanoemulsion agent has been shown to be internalized by immune cells in multiple studies^12,20,29,30,36,39^, including T cells^29^.

Immune cell labeling with the nanoemulsion used (CS-1000) does not alter cell viability, phenotype or cytokine production^23,30,31,36^. In addition, the same imaging agent has been used in a clinical trial monitoring a PFC-labeled dendritic cell immunotherapy in colorectal cancer patients^7^. Moreover, CS-1000 is the subject of an FDA Drug Master File (DMF), as well as a similar DMF at Health Canada. Thus, the safety of this PFC nanoemulsion to cells and organisms at appropriate doses used in cell therapy has been extensively investigated. Utmost care and caution should nonetheless be applied before proceeding with any *in vivo* application of ^19^F-labeled CAR T cells in human subjects.

Alternative methods for assessing cell distribution in preclinical studies often involve laborious and tissue-destructive histopathology, or flow cytometry measurements in blood and tissue samples prepared as single cell suspensions^3,40,41^. Analyses of tissues requires multiple manual processing and chemical treatment steps, as well as human input in the data collection and computer analysis, all of which are time-intensive. Histopathology remains semi-quantitative for analysis of multi-organ tissue panels, and is often limited by a finite number of tissue slices leading to potential sampling error, as well as confounding factors such as tissue autofluorescence^42^. Flow cytometry is most often used to measure remaining circulating T cells in blood. Others have reported that the average LOD for circulating T cells by flow cytometry is on the order of 3–10 CAR T cells per microliter of blood^41,43,44^. Thus, flow cytometry, as well as histology, may enable the detection of ‘rare’ cells in tissues.

To conclude, ^19^F NMR cytometry is a rapid and quantitative technique to evaluate adoptive cell transfer biodistribution in intact tissues. This technique provides unbiased quantification in samples. Overall, ^19^F NMR cytometry may accelerate the timeline to evaluate new immunotherapeutic cell candidates by providing a straightforward method to evaluate cell therapy biodistribution and cell fate.

## Materials and Methods

### Chimeric antigen receptor (CAR) lentiviral vector

We employed a vector for CAR consisting of a side chain fragment variable (scFv) specific to EGFR-vIII fused to a CD8α hinge with 4-1BB, CD28 and CD3z intracellular domains as described by Ohno *et al*.^45^. With this construct, lentivirus was produced using ten million human embryonic kidney (HEK) 293 T cells (ATCC, Manassas, VA) that were plated in a 175 cm^2^ flask with 9 ml of OptiMEM (Gibco) media. The psPAX2, pMD2.G and pELNS-3C10CD28–41BBz plasmids were added to the flask with 120 μl of Lipofectamine (Invitrogen, Carlsbad, CA) as described elsewhere^45^. After 24 hours, the supernatant was harvested and centrifuged at 16,000 rpm for 2 hours at 4 °C. The viral pellet was resuspended in 100 μl of phosphate buffered saline (PBS) and used immediately to transduce human T cells (described below).

### Human T cell isolation

Peripheral blood mononuclear cells (PBMCs) were isolated from anonymous donor human blood (San Diego Blood Bank, San Diego, CA) by Ficoll (Histopaque 1077, Sigma Aldrich, St Louis, MO) gradient density centrifugation. T cell enrichment from PBMCs was performed by Pan T cell magnetic cell sorting (Miltenyi Biotech Inc., Auburn, CA). Cell phenotype was confirmed by flow cytometry (LSR Fortessa, BD Biosciences, San Diego, CA) using FITC anti-human CD3 antibody (Biolegend, San Diego, CA). T cells were expanded for two days in Roswell Park Memorial Institute (RPMI) media (Gibco) supplemented with 10% Fetal Bovine Serum (FBS) and 100 units/ml of recombinant human Intereukin 2 (IL-2, Peprotech, Rocky Hill, NJ) while being activated with dynabeads harboring human T cell CD3/CD28 (Gibco).

### CAR transduction and PFC labeling

The T cells were added to 6-well plates at a density of 1 × 10^6^ cells per well and transduced with CAR lentivirus; 30 μl of virus in PBS was added to each well along with 6 μg/ml Polybrene transfection agent (EMD Millipore, Billerica, MA). The T cell transduction efficacy was determined by flow cytometry at 5 and 14 days after virus addition using a primary biotin-SP-AffiniPure F(ab’)2 fragment-specific goat anti-mouse antibody (Jackson Immuno Research Laboratories, West Grove, PA) and streptavidin-PE served as the secondary antibody (BD Pharmingen, San Diego, CA).

To label T cells for NMR cytometry, CAR T cells and untransduced T cells (control) were plated at a density of 10 million cells in 5 ml of RPMI in 6-well plates and incubated for 12 h with 10 mg/ml of PFC nanoemulsion (CS-1000 or CS-ATM DM Red, Celsense, Inc., Pittsburgh, PA). Controls (unlabeled) cells were plated in identical conditions with no label added. Subsequently, labeled cells were washed three times with PBS to remove excess emulsion and assayed for viability via the Trypan blue assay. In controls cells, the same PFC volume was added, without the incubation step, after the viability measurement and washed three times with PBS. ^19^F NMR spectra of control cell pellets did not show residual PFC emulsion, confirming that non-internalized PFC does not pellet during the wash steps^46^.

### Flow cytometry assays

CD4/CD8 phenotype and viability was also validated by flow cytometry using PE/Cy5 anti-human CD4 clone OKT4, Alexa 488 anti-human CD8 clone SK1 and 7-AAD viability marker (Biolegend). In these assays, T cells minus PFC label, with and without CAR transgene, were evaluated alongside as controls. Potential impact of PFC labeling was also evaluated by measuring CD4, CD8, and 7-AAD viability marker by flow as described above, as well as CAR T cell proliferation rate, at day 2, 7 and 14 post-labeling and compared to unlabeled control. Persistence of dual-mode PFC (CS-ATM-DM-Red) label in CAR T cells was assessed by flow cytometry over 14 days after wash.

### Confocal microscopy

Aliquots of CAR T cells (N = 6, 1 million cells) labeled with dual-mode PFC emulsion or unlabeled were harvested one day post-labeling, fixed with 4% paraformaldehyde (Affimetrix Inc., Cleveland, OH) for 10 min and stained with CD3-FITC as above and Hoechst dye nuclear stain (#33342, 1:500 dilution, Thermo Fisher Scientific). Cells were mounted in media (Lerner Laboratories, Cheshire, WA) and slides were imaged using a confocal Leica DM 6000 CFS microscope with a ×63 immersion objective.

To quantitatively assay PFC cell uptake, triplicates of 1.5 million cells were resuspended in 150 μl PBS with 0.5% Triton X-100 (Sigma Aldrich) and transferred to a 5 mm NMR tube. Also, a 50 μl solution of 0.1% sodium trifluoroacetate (TFA, Sigma Aldrich) in D_2_O (Acros Organics, Geel, Belgium) was added to each tube and vortexed. ^19^F NMR spectra were acquired for each sample using a 400 MHz (9.4 Tesla) Bruker AVANCE III HD-NanoBay spectrometer (Bruker, Inc., Billerica, MA) with: 17 μs pulse, 32,000 FID points, 100 ppm spectral width, 32 averages, and a recycle delay of 5 s. Two peaks were observed, with the TFA reference and PFC peaks at -76 ppm and -91.58 ppm, respectively. The ^19^F content of each sample was determined by calculating the ratio of the PFC peak integrated area to the reference integral multiplied by the number of fluorine atoms in the TFA aliquot. The mean ^19^F/cell was calculated from the ^19^F content divided by the cell count in the sample.

### Electron microscopy

We examined CAR T cells labeled with PFC nanoemulsion by electron microscopy. Pelleted cells were fixed in PBS containing 2% glutaraldehyde in 0.1 M sodium cacodylate (SC) buffer at room temperature for 30 min and held overnight at 4 °C. The cells were washed five times in 0.1 M SC buffer on ice and treated with 1% OsO_4_ in 0.1 M SC buffer for 1 hour. All of the samples were washed in dH_2_O and treated with 2% uranyl acetate for 1 hour on ice. Pellets were then dehydrated in an ascending series of ethanol and finally 2× dry acetone at room temperature. The cells were infiltrated overnight in a solution containing a 1:1 mixture of dry acetone and Durcupan for 2 hours on a rotator and in 100% Durcupan overnight. The next day, the mixture was replaced with 100% Durpucan twice, and pellets were embedded in Durpucan and incubated in an oven for 36–48 hours. Ultra-thin (60 nm) sections were cut using a diamond knife and collected on mesh Cu grids. The samples were stained with 1% aqueous uranyl acetate and Reynolds lead citrate. Sections were imaged using a Tecnai Spirit electron microscope at 80KV (FEI company, Hillsboro, OR).

### Glioblastoma cells

Frozen U87-EGFRvIII-Luc glioblastoma cells^45^ overexpressing EGFRvIII, as well as the luciferase gene, were thawed and maintained in RPMI media containing 10% FBS and 1% penicillin/streptomycin (Gibco) in T75 flasks (Sigma).

### *In vitro* T cell cytotoxicity assay

U87-EGFRvIII-Luc cells were plated at a density of 30,000 cells per well (18 wells total) in clear bottom 96-well plates (Corning, Inc., Corning, NY) and were allowed to adhere. Wells (n = 6, per condition) received: (i) 5:1 ratio of CAR T cells to cancer cells, (ii) 5:1 ratio of untransduced T cells to cancer cells, or (iii) cancer cells alone to calculate baseline viability. Of note, CAR T and untransduced T cells used in this experiment were initially stimulated with CD3/28 beads without re-stimulation for several days prior to performing the cytotoxicity assay. After adding D-luciferin (300 μg/ml) to each well, bioluminescence signals were measured with a Tecan plate reader (Infinite M200PRO, Morrisville, NC) at 6, 12, and 24 hours post T cell addition. The relative glioma cell cytotoxicity was obtained from the mean photon count for groups (i-iii), and the ratio of treated versus untreated means are displayed as percentages.

### Murine model of subcutaneous glioblastoma

Animal protocols were approved by the University of California San Diego Institutional Animal Care and Use Committee (IACUC) and experiments were performed in accordance with IACUC guidelines and regulations. *In vivo* studies were performed to confirm anti-cancer efficacy of PFC-labeled CAR T cells. Female (n = 45) SCID mice 6–8 weeks of age (Jackson Laboratories, Bar Harbor, ME) received bilateral sub-cutaneous flank injections, each containing 5 × 10^6^ U87-EGFRvIII-Luc cells in buffered 50% Matrigel (Corning, Tewksbury, MA). Once tumors were established (~4 mm size), mice were divided into 3 groups (n = 15 per group), with Group 1 receiving 20 × 10^6^ PFC labeled CAR T cells intravenously (IV) via tail vein, Group 2 receiving the same number of PFC-labeled untransduced T cells IV, and Group 3 was untreated. For histology purposes, one additional animal in Groups 1–2 received T cells labeled with a fluorescent dye-conjugated PFC nanoemulsion (CS-ATM DM Red, ex/em 596/615, Celsense Inc.), to enable histological validation of colocalization of imaging agent and transferred labeled T cells.

### *In vivo* bioluminescence imaging (BLI)

All mice underwent serial BLI using an IVIS Spectrum (Perkin Elmer, Waltham, MA) at day 0, 3, 7, 10 and 14 after receiving T cells. Prior to BLI, mice received 150 mg/kg of D-Luciferin (Intrace Medical, Lauzanne, Switzerland) intraperitoneally, and images were acquired 10 to 15 min after injection to measure total flux. A white-light body surface image was collected with a field of view to fit five mice, followed by an image of the spatial distribution of photon counts rendered in pseudo-color, which was overlaid onto the surface image. Quantitative analysis of the radiance flux (photons/s) was performed with the Living Image Software (Perkin Elmer, Waltham, MA) by defining identical regions of interest covering the tumor area. Following BLI, tumor sizes were measured using a caliper.

### NMR cytometry limits of detection (LOD)

We assessed the LOD for labeled T cells on a standard 400 MHz NMR spectrometer. Exchangeable 5 mm and 10 mm sample-NMR probes (Bruker models BBFO and BBO, respectively) were evaluated. Sample standards with varying ^19^F content were prepared from serial dilutions of 0.05 to 0.5% TFA/D_2_O in volumes of 250 or 1,500 μl for 5 or 10 mm probes, respectively. All sample spins were contained within the receptive field of the NMR probe. The ^19^F NMR spectra were acquired using: 17 μs pulse, 32,000 points acquired for free induction decay (FID), 100 ppm spectral width, 128 averages, 20 min acquisition time, and a recycle delay of 10 s. The signal-to-noise ratio (SNR) of the TFA spectra were calculated using TopSpin software (Bruker). The results were plotted as the SNR versus ^19^F content measured for each NMR sample probe (5 or 10 mm), and LOD was defined as the ^19^F atom count where the extrapolated SNR = 2.

### *Ex vivo*^19^F NMR

In each animal group, five mice were euthanized at days 2, 7 or 14 after treatment infusion to evaluate cell biodistribution. Mice were sacrificed by CO_2_ inhalation, followed by blood removal using a syringe and cardiac puncture. Intact tissues of interest (brain, thymus, heart, lungs, liver, spleen, tumors, lymph nodes, kidneys, small intestine, spinal cord, femur, tail and blood) were harvested. All organs were fixed in 4% paraformaldehyde overnight, rinsed in PBS and weighed before transfer to either 5 or 10 mm glass NMR tubes (Wilman Labglass, Vineland, NJ), depending on organ size. Blood collected by cardiac puncture was also measured by NMR. Importantly, the specimen size/blood volume fit entirely within the manufacturer-specified receptive field of the NMR detector coil, which spanned ~2 cm length from the bottom of the NMR tube, thereby ensuring that all ^19^F nuclei in the sample are detected. Also, a sealed glass reference capillary (Kimble Kontes, Vineland, NJ) containing a mixture of 0.1% (w/v) TFA, as well as 0.325 mM MnCl_2_ to shorten the TFA’s ^19^F T_1_ relaxation to match that of the PFC nanoemulsion (T_1_~470 ms at 9.4 Tesla)^47^, was placed inside the NMR tube with the sample. All samples were first measured individually, and for organs/tissues with very low ^19^F signal, multiple specimens were pooled into a single NMR tube to boost sensitivity. After shimming, the ^19^F NMR spectra were acquired using a 400 MHz spectrometer with: 17 μs pulse, 32,000 FID points, 100 ppm spectral width, 32 to 1024 averages (depending on SNR), and a recycle delay of 1.5 s. Phase and baseline corrections were performed to improve measurement accuracy. The ^19^F content of each tissue sample was determined by calculating the ratio of the PFC peak (−91.58 ppm) integrated area to the TFA reference (−76 ppm) integral, multiplied by the number of fluorine atoms in the reference. The apparent cell number per tissue sample was calculated by dividing the ^19^F content of each tissue sample by the mean ^19^F/cell of T cells measured after labeling (above).

### Histological analyses

In the one animal per experimental group receiving fluorescent PFC nanoemulsion (CS-ATM DM Red, Celsense) spleens, tumors and livers were embedded in optimal cutting temperature (OCT) compound (Sakura Finetek USA, Inc., Torrance, CA) and stored at −80 °C. Additional animals (N = 2 receiving CAR T cells and N = 2 receiving untransduced T cells) were sacrificed at day 2 for histopathology purposes. All tissues were cryosectioned (CM1950, Leica Microsystems Inc., Buffalo Grove, IL) at 10 μm thickness. Sections were fixed with 4% paraformaldehyde, stained for T cells using FITC anti-human CD3 (UCHT1, 1:500 dilution, Biolegend) and for nuclei using Hoechst dye (1:500) and then mounted. Tumor, spleen and liver sections were stained for macrophages with an Alexa 488 anti-mouse F4/80 antibody (BM8, 1:200 dilution, Biolegend)^48^. Fluorescence images were acquired on an Axiovert 40 CFL microscope (Zeiss, Thornwood, NY) using a ×5 objective. Confocal images were acquired on a Leica SP5 2 confocal system with a Leica DM 6000 CFS microscope and a ×63 immersion objective. For direct cell counts in tumor, we used sections (two per tumor) stained against CD3 from five tumors total, three from day 2 and one each from days 7 and 14. We counted T cells in six high power fields per slice (×20 magnification, 120 high power fields total).

### Statistical analyses

Measurements are presented as mean ± standard error. A one-way ANOVA along with unpaired T-tests with unequal variances were performed to compare groups as a whole and by pairs, respectively, for bioluminescence and tumor volume measurements. For ANOVAs, we used the Bonferroni correction for multiple comparisons, thereby controlling the family-wise error rate at 5%. Unpaired T-tests were used to compare the doubling time, viability, apparent cell number per organ, and high power field T cell count between groups. P-values < 0.05 are considered statistically significant. ANOVA results are expressed as an F-statistic and its associated degrees of freedom and P-value. For pooled samples (thymus and lymph nodes), an estimate of the NMR measurement error is displayed, which was calculated from the standard error of the baseline noise signal over the same integral interval width used to measure the ^19^F tissue peak. Average ^19^F NMR signal in tumors was correlated with the number of CAR or untransduced T cells count by histopathology using a Pearson’s correlation coefficient test.

### Data availability

The data that support the findings of this study are available from the corresponding author upon request.

## Electronic supplementary material

Figure S1 and Table 1
