# Supplementary material for: Fluorine-19 nuclear magnetic resonance of chimeric antigen receptor T cell biodistribution in murine cancer model
Source: Sci Rep. 2017 Dec 18;7:17748. doi: 10.1038/s41598-017-17669-4 (PMC5735180; doi:10.1038/s41598-017-17669-4)
Supplement: Supplementary file 1 — Figure S1 and Table 1 [file 41598_2017_17669_MOESM1_ESM.docx]

**Fluorine-19 nuclear magnetic resonance cytometry to quantify chimeric antigen receptor T cell biodistribution in murine cancer model**

Fanny Chapelin, Shang Gao, Hideho Okada, Thomas G. Weber, Karen Messer, Eric T. Ahrens

**SUPPLEMENTARY INFORMATION**

Supplementary figure S1:


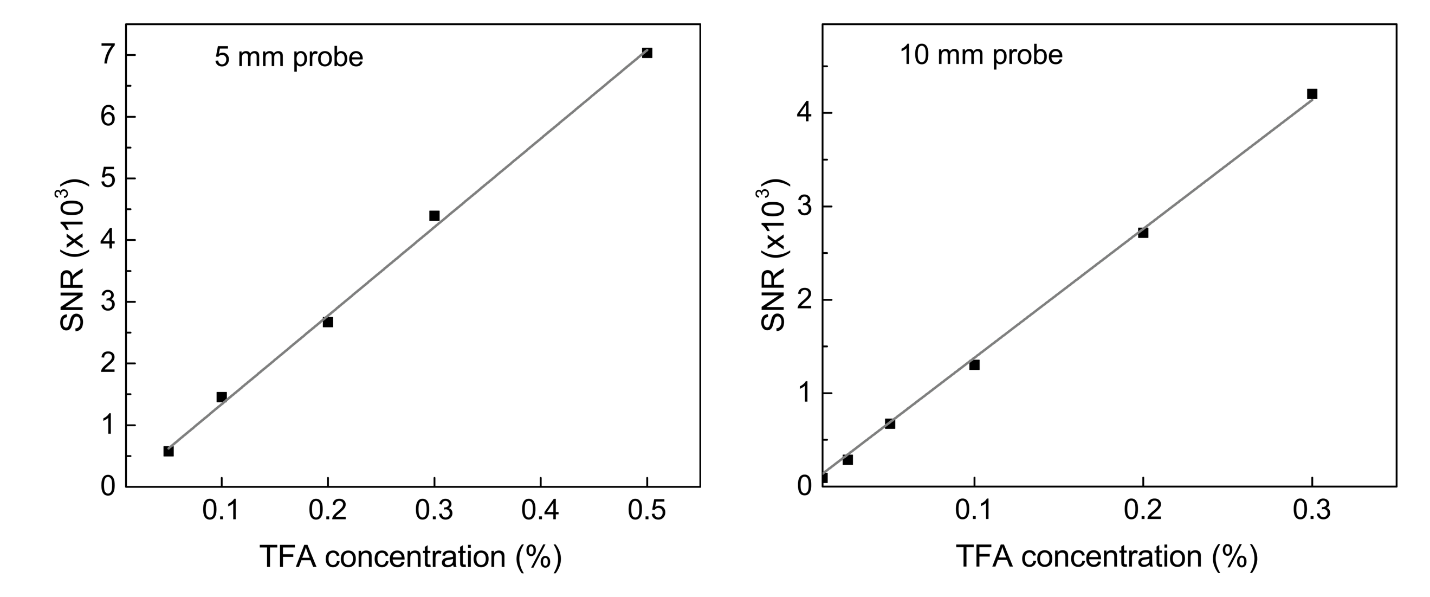


**Figure S1.** **Fluorine-19** **NMR limit of detection (LOD) estimation.** Panels display the signal to noise ratio (SNR) as a function of fluorine concentration in sodium trifluoroacetate (TFA) reference for a standard 5 and 10 mm probe (left and right, respectively). After a linear fit, the LOD is defined as the ^19^F atom count where the extrapolated SNR=2. The results yield a LOD of approximately ~ 10^14^ and ~10^15^ ^19^F atoms for the 5 and 10 mm probe respectively, corresponding to ~7×10^3^ and ~4×10^4^ cells assuming typical ^19^F labeling levels in T cells. All ^19^F NMR data were acquired on at 376.3 MHz with a 20 min acquisition time and using a 17 μs pulse, 32,000 points of free induction decay (FID), 100 ppm spectral width, 128 averages, and recycle delay of 10 s.

Table 1:


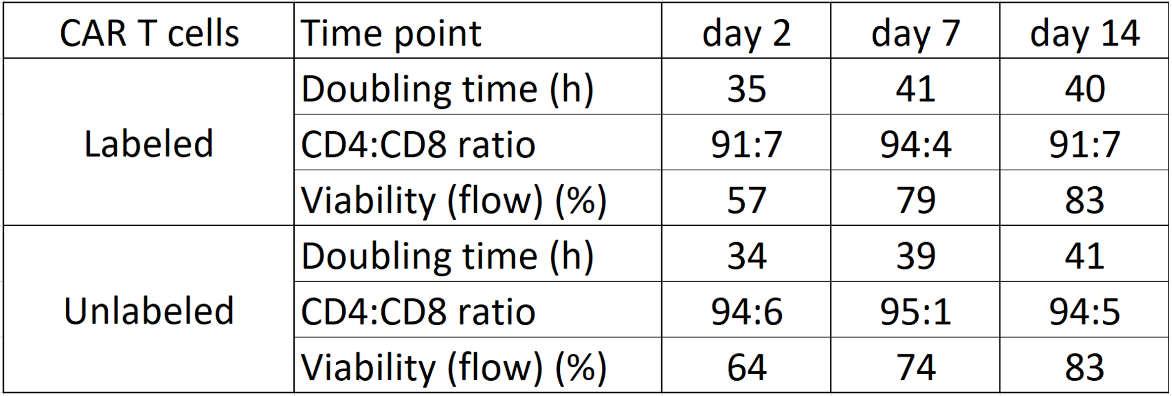


**Table 1. Longitudinal characterization of PFC-labeled CAR T cells.** Table displays average doubling time, CD4/CD8 ratio and viability as measured by flow cytometry of PFC labeled CAR T cells and control unlabeled CAR T cells at days 2, 7 and 14 after PFC labeling (N=3 replicates). No significant differences were seen between labeled and unlabeled CAR T cells for all criteria.
